# Renal Cell Carcinoma with Intraluminal Spread of the Entire Upper Urinary Tract

**DOI:** 10.1155/2013/371387

**Published:** 2013-05-07

**Authors:** Shigenori Kakutani, Haruki Kume, Yoshikazu Hirano, Toshihiko Wakita, Yukio Homma

**Affiliations:** ^1^Department of Urology, The University of Tokyo Hospital, Hongou 7-3-1, Bunkyo-ku, Tokyo 1138655, Japan; ^2^Department of Urology, Fraternity Memorial Hospital, Yokoami 2-1-11, Sumida-ku, Tokyo 1308587, Japan; ^3^Department of Radiology, Fraternity Memorial Hospital, Yokoami 2-1-11, Sumida-ku, Tokyo 1308587, Japan

## Abstract

We describe an unusual case of renal cell carcinoma (RCC) involving the entire upper urinary tract. A 51-year-old female was referred to us because of macroscopic hematuria. Computed tomography revealed a renal tumor filling renal pelvis and ureter, which turned to be a clear cell RCC after nephroureterectomy.

## 1. Introduction

Although RCC occasionally invades the renal pelvis and ureter microscopically, massive extension into the renal pelvis and ureter is a rare event. Herein, we describe a case with an RCC growing in the renal pelvis and ureter and reaching the bladder.

## 2. Case Presentation 

**Figure 1:**
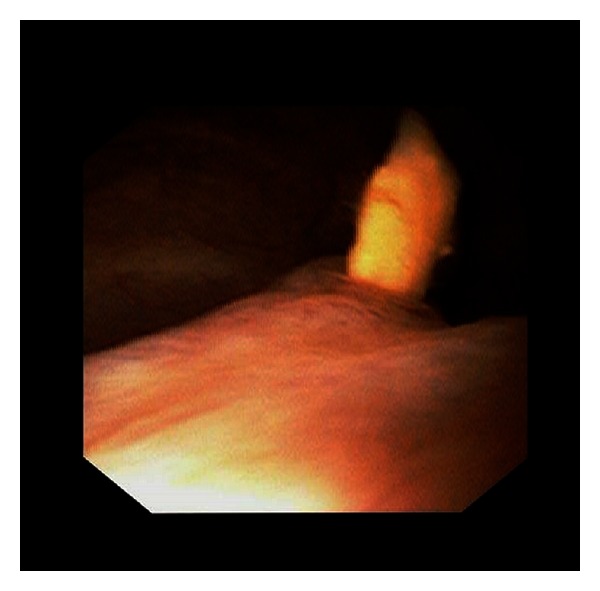
Cystoscopy showed the tumor emanating from the left ureteral orifice.

**Figure 2:**
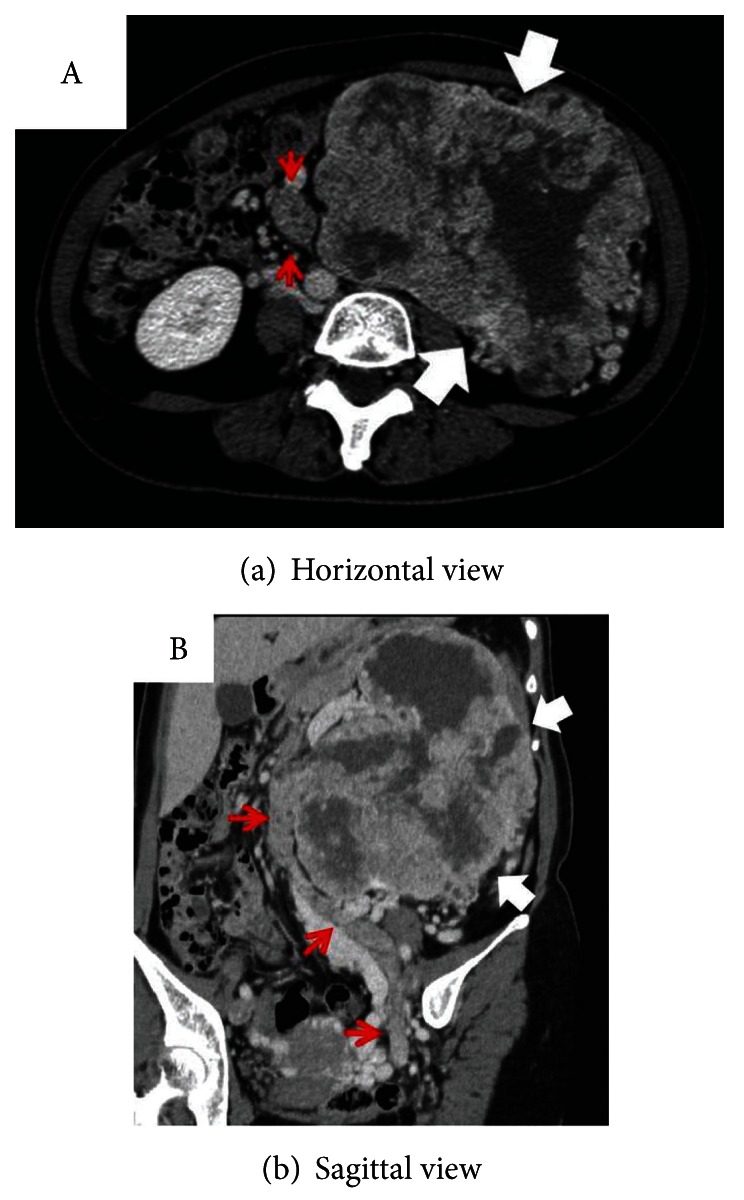
Contrast enhanced CT showed a left renal tumor with heterogenous enhancement (white arrows). Left ureter was packed with the tumor (red arrows).

**Figure 3:**
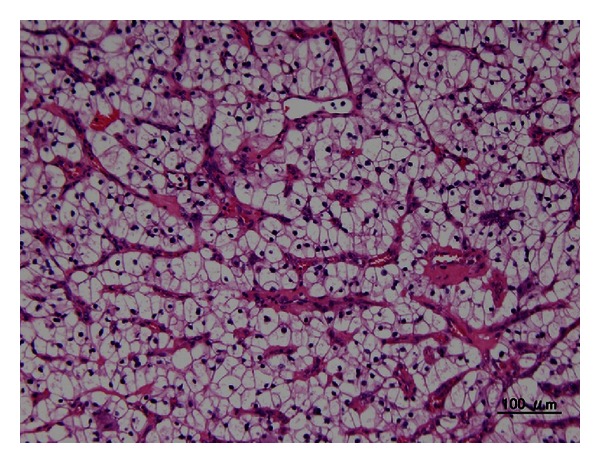
Histological examination revealed that the tumor was clear cell RCC, G2 > 1, with microvascular invasion.

A 51-year-old female visited us due to asymptomatic macroscopic hematuria persisting for two weeks. She had experienced discomfort, not pain, in the stomach. She had been followed up at another clinic for polycythemia for several years. Cystoscopy revealed a nonpapillary tumor about 2 cm in diameter, emanating from the left ureteral orifice (Figure 1). Blood results at presentation showed Hb 14.8 g/dL, Cr 0.7 mg/dL, estimated GFR 68.1 mL/min, WBC 6.6 × 10^3^/*μ*L, CRP 5.35 mg/dL, LDH 184 IU/L, and Ca^2+^ 9.9 mg/dL. Urine cytology was negative for malignant cells.Contrast enhanced CT showed an unevenly contrasted tumor, infiltrating almost all renal parenchyma, filling the renal pelvis and ureter, and extending into the urinary bladder (Figure 2). There were no apparent metastases to lung, liver, adrenal glands, or lymph nodes. With a tentative diagnosis of renal pelvic tumor, we didnot perform perioperative biopsy, and she underwent nephroureterectomy with hilar lymph node dissection. Macroscopically the tumor almost replaced the whole kidney. The renal pelvis and ureter were extensively packed with the tumor. Histological examination (Figure 3) revealed a clear cell RCC, grade 2 > 1, with microvascular invasion, Fuhrman grade 2, without any sarcomatoid elements. There was no evidence of a venous thrombus or metastasis to lymph nodes. Leibovich score was 6. Postoperative course was uneventful, but 6 months later multiple pulmonary metastases were detected.

## 3. Discussion

Although RCC may penetrate the renal pelvis, intraluminal spread into the renal pelvis and ureter is extremely rare with only a few cases reported in the English literature [1–6]. Munechika et al. [3], Chen et al. [4], and Fujita et al. [5] reported similar cases, although the tumor extension was limited in the renal pelvis and ureter. Gulati et al. [6] reported a case in which the tumor protruding from the ureteral orifice was resected transurethrally and confirmed as a clear cell RCC. Interestingly, in these cases, as well as ours, no visible venous thrombus was reported. We did not resect the tumor in the bladder transurethrally because we considered it was less likely lymphoma, whose tumor marker, soluble IL-2R, was 643 U/mL a little over normal range.

Also, enhanced CT findings did not show hypovascular pattern, suggesting that it would not be sarcoma.

Intraluminal spread of the upper urinary tract by other types of cancer has also been documented (Table 1). Thorup reported a case of implantation of colonic adenocarcinoma which occluded the ureter a year after the surgery with ureteral injury. Tsurumaki et al. described a case of uterine endometrioid carcinoma filling the upper urinary tract 11 years after hysterectomy with partial ureterectomy for invasion.

The implantation and/or invasion to urothelial mucosa followed by intraluminal expansive growth would be the pathogenesis of this rare manifestation. However, because of the rarity of these cases, clinical characteristics have not been fully understood.

## Figures and Tables

**Table 1:** 

Author (year)	Age/sex	Primary disease	Time to metastasis	Which regions of the ureter	Operation
Williams and Chaffey, l966 [7]	69/M	Adenocarcinoma of the sigmoid colon	4 years	Lower third of bilateral ureters	Bilateral nephrostomy
Thorup et al., 2001 [8]	76/M	Adenocarcinoma of the sigmoid colon	1 year	Lower left ureter	Partial ureterectomy
Tsurumaki et al., 2009 [9]	72/F	Uterine endometrioid carcinoma	11 years	From the left pelvis to the pelvic ureter	Nephroureterectomy
